# The Tooth-Crown Patents

**Published:** 1887-04

**Authors:** 


### THE TOOTH-CROWN PATENTS

A decision has been rendered in the patent suit of the International Tooth-Crown Company *vs.* C. M. Richmond *et al.* A victory is claimed by both parties. The case was tried in the United States Circuit Court for the Southern District of New York, before Justices Wallace and Shipman. The opinion is by the former, and commences: "The complainant is the owner of four patents relating to improvements in the dental art, all of which are alleged to be infringed by the defendants. This suit is brought for an injunction and accounting."

The opinion sums up as follows: "A decree is ordered for an injunction and an accounting as to the first of the patents in suit. As to the others, the bill is dismissed. Neither party is awarded costs."

Dentists will naturally desire to know what are the four patents involved in the suit. The opinion states that the first was granted to James E. Low, March 15, 1881, and is "an improvement in dentistry whereby artificial dental surfaces may be permanently fixed in the mouth in place of lost teeth without the use of plates or other means of deriving support from the gum beneath the artificial dentition." The patent itself says:—

"What I now claim as new is—

"1. The herein described method of inserting and supporting artificial teeth, which consists in attaching said artificial teeth to continuous bands fitted and cemented to the adjoining permanent teeth, whereby said artificial teeth are supported by said permanent teeth without dependence upon the gum beneath.

"2. An artificial tooth cut away at the back, so as not to present any contact with the gum except along its front lower edge, and supported by rigid attachment to one or more adjoining permanent teeth, substantially as and for the purpose set forth."

The second patent in suit was granted May 22, 1883, to Cassius M. Richmond, assignor, etc., and is for the invention known in the dental profession as the Richmond Tooth Crown. The decision declares this patent invalid.

The third patent was granted to Alvan S. Richmond, May 22, 1883, for an artificial denture, and is also declared invalid.

The fourth patent is declared invalid for want of novelty.

As we understand it, the dentist who attaches a bridge of teeth to bands fitted and cemented to adjoining permanent teeth and supported by such teeth, infringes the Low patent, which is sustained by the decision, and such dentist is liable for damages unless he shall have secured the consent of the International Tooth-Crown Company. The claim is for a *permanent* bridge, cemented to adjoining teeth. It would not seem, then, if we rightly understand the claim, that any other kind of bridge is not covered by the patent. If it be anchored in any way other than by "continuous bands fitted and cemented to the adjoining permanent teeth," we cannot see that it would be an infringement. If it be a removable bridge, the claim would not seem to cover it. The bridge, as usually constructed and cemented to adjoining teeth, is not free to the public, but unless the decision is overruled by some higher court, will stand as the property of the International Tooth-Crown Company.

The patents for the Tooth-Crowns, at least all those adjudicated upon, if we can understand a law paper correctly, are henceforth, so long as this decision shall stand, free to the public, and may be used by any one.

